# Coupled Criticality Analysis of Inflation and Unemployment

**DOI:** 10.3390/e23010042

**Published:** 2020-12-30

**Authors:** Zahra Koohi Lai, Ali Namaki, Ali Hosseiny, Gholamreza Jafari, Marcel Ausloos

**Affiliations:** 1 Department of Physics, Islamic Azad University, Firoozkooh Branch, Firoozkooh 3981838381, Iran; z_koohilay@yahoo.com; 2 Department of Finance, Faculty of Management, University of Tehran, Tehran 1411713114, Iran; 3 Iran Finance Association, Tehran 1411713114, Iran; 4 Department of Physics, Shahid Beheshti University, Tehran 1983969411, Iran; al_hosseiny@sbu.ac.ir (A.H.); g_jafari@sbu.ac.ir (G.J.); 5 Center for Network Science, Central European University, 1051 Budapest, Hungary; 6 School of Business, College of Social Sciences, Arts, and Humanities, Brookfield, University of Leicester, Leicester LE2 1RQ, UK; ma683@le.ac.uk; 7 Group of Researchers for Applications of Physics in Economy and Sociology (GRAPES), Rue de la belle jardinière, 483, Sart Tilman, Angleur, B-4031 Liege, Belgium; 8 Department of Statistics and Econometrics, Bucharest University of Economic Studies, Calea Dorobantilor 15–17, Sector 1, 010552 Bucharest, Romania

**Keywords:** non-Gaussianity, bivariate multifractal random walk, inflation, unemployment, complex systems

## Abstract

In this paper, we focus on the critical periods in the economy that are characterized by unusual and large fluctuations in macroeconomic indicators, like those measuring inflation and unemployment. We analyze U.S. data for 70 years from 1948 until 2018. To capture their fluctuation essence, we concentrate on the non-Gaussianity of their distributions. We investigate how the non-Gaussianity of these variables affects the coupling structure of them. We distinguish “regular” from “rare” events, in calculating the correlation coefficient, emphasizing that both cases might lead to a different response of the economy. Through the “multifractal random wall” model, one can see that the non-Gaussianity depends on time scales. The non-Gaussianity of unemployment is noticeable only for periods shorter than one year; for longer periods, the fluctuation distribution tends to a Gaussian behavior. In contrast, the non-Gaussianities of inflation fluctuations persist for all time scales. We observe through the “bivariate multifractal random walk” that despite the inflation features, the non-Gaussianity of the coupled structure is finite for scales less than one year, drops for periods larger than one year, and becomes small for scales greater than two years. This means that the footprint of the monetary policies intentionally influencing the inflation and unemployment couple is observed only for time horizons smaller than two years. Finally, to improve some understanding of the effect of rare events, we calculate high moments of the variables’ increments for various *q* orders and various time scales. The results show that coupling with high moments sharply increases during crises.

## 1. Introduction

Unemployment and inflation are two important economic features; their relation is highly meaningful for policymakers. It is common knowledge of experts that the possible relation that holds between these two variables has been a key controversial issue among different schools of economic thought.

Indeed, historically, this controversy flared up with the observation of Alban W. Phillips’ report on a negative relationship between unemployment and the growth of wages from 1861 until 1957 in the United Kingdom [1]. Such a negative relation was named after him, i.e., the Phillips curve [2].

A few months after the publication of A.W. Phillips, Robert Solow, and Paul Samuelson published related findings. They observed a similar negative correlation between unemployment and inflation in the United States [3]. These findings begot an illusion that policymakers can permanently decrease unemployment at the cost of high inflation. Warnings about such a policy however were made by the new classical economists such as Milton Friedman and Edmund Phelps [2,4,5,6]. They argued that if, in the long run, policymakers increase the volume of money, agents realize the policy and increase prices. As a consequence of these situations, price growth would not be accompanied by a decline in unemployment, as wished.

In the 1970s, the prediction by these new classical economists came true. A long-lasting period of stagnation started during which expansionary monetary policies failed to downturn unemployment; a positive relationship between unemployment and inflation emerged during a decade [7].

Currently, in the mainstream, one believes that the growth of money cannot boost the economy and decrease unemployment if one imposes a sustained and permanent inflation policy. If policymakers aim to let the money volume increase permanently, agents become so accustomed that the government policies become ineffective in the long run. Nevertheless, the Federal Reserve looks at the volume of money and at the interest rate as apparatuses, even levers, to influence the economy in the short run. In other words, policymakers at the Federal Reserve believe that unexpected changes in the volume of money influence economic conditions in the short run. However, an interesting question pertains to the time scale that distinguishes the short run from the long run relationship between inflation and unemployment.

Thus, the true relation between unemployment and inflation and especially the causality direction between them are still serious controversial issues in economics; see for example [8,9,10,11,12,13,14,15,16,17,18,19] and the references therein. The debate especially finds a crucial meaning if policymakers aim to impose expansionary monetary policies to boost production during recessions. There is no need to observe that this controversy is a quite pertinent dilemma at the time of writing (during the COVID-19 pandemic) [20,21].

Yet, few works have considered the so-called theoretical complexity of these variables. This gap thereby leads to the research questions of this paper, as in [22,23].

On the other hand, complexity economics has attracted a great deal of attention in recent years [24,25,26,27,28,29,30,31,32,33,34,35]. Indeed, the economy can be considered as a huge network of heterogeneous agents who interact with each other and with their environment [34,36,37,38,39,40,41,42,43]. It is reasonable to expect that inflation and unemployment, as outcomes of these complex systems, inherent complexity theory features. This suggests considering a thorough analysis of these variables along with advanced techniques available in the complexity theory approach, itself found to be of interest in many applications [44,45,46,47].

Looking at unemployment and inflation indices as simple variables forces one to ignore some rich knowledge about their complexity. It has been shown for example that economic indicators and their coupling have nice scaling and present multifractal features [22,48,49,50,51,52]. Scaling analysis has proven successful to address a wide range of phenomena in nature; see for example [53,54,55,56,57,58]. Economic indices are not an exception [49,50,59,60,61,62]. Moreover, these multiscaling features can be also described through an entropy function and free energy as introduced for multifractals [63,64,65].

In this work, to grab scaling features of unemployment and inflation, and to analyze the role of large fluctuations, we focus on the non-Gaussianities in their probability density functions (PDF). For illustration, we focus on monthly records of inflation and unemployment rates, provided by the U.S. Inflation Calculator [66] and U.S. Bureau of Labor Statistics [67], for the period lasting from January 1948 until October 2018 [68].

**Figure 1:**
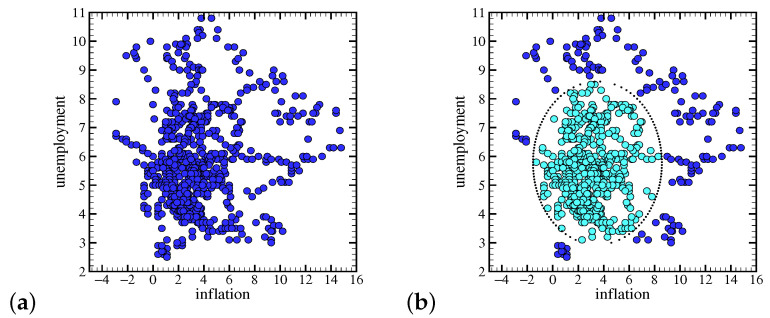
Unemployment vs. inflation. Each point represents the unemployment vs. inflation rate. The inflation rate is the year-on-year consumer price index (CPI) reported monthly for the period ranging from January 1948 till October 2018; (**a**) All data points, 850. (**b**) Regular events (greenish blue points, 702 points) and the rare events (dark blue, 148 points) via an ellipse. The center of this ellipse has to coordinate the mean value of each data. Each chord of the ellipse in whatever direction is three times the standard deviation of the data in that direction. This means that points outside the ellipse are three standard deviations away from the mean value. Source: U.S. Inflation Calculator [66] and Bureau of Labor Statistics [67].

Figure 1a illustrates such monthly data during the period. The mean (standard deviation) % values of inflation and unemployment for this term are 3.51(2.93) and 5.76(1.64), respectively. The correlation coefficient between unemployment and inflation for the whole data depicted in Figure 1 is less than ∼0.05, which is not statistically meaningful.

Figure 1b shows two areas for the data points: “regular” and “rare” events. Regular events are those events that are inside an ellipse centered on the mean value coordinates of both data with a “chord” of three standard deviations (3σ) in each possible direction in the plane. In other words, we consider that the rare events are those events that are at least 3σ away from the mean value.

If we exclude regular events and focus on the tail of the distribution illustrated in Figure 1b, then the correlation coefficient is ≃−0.24. This means that the response of the economy to rare events might be different from regular events.

## 2. Methods

### 2.1. Multifractal Random Walk (MRW)

In essence, the Multifractal Random Walk (MRW) is used to analyze time series stems from turbulent cascade models [69]. MRW is a model to study multifractality based on the non-Gaussianity of the PDF. The model generates a non-Gaussian time series of fluctuations through protecting two-time series, a Gaussian and a log-normal, such that the non-Gaussian parameter is the standard deviation of the log-normal signal. One can show that in an MRW, the non-Gaussianity parameter is related to multifractality features [56,70].

Thus, MRW processes are useful for representing the non-Gaussian behavior of time series. Practically, the temporal fluctuations increment of a process at scale *s*, $\delta_{s}x\left(t\right)=x\left(t+s\right)-x\left(t\right)$, are modeled by the product of a normal and a log-normal process:

(1) $$\delta_{s}x\left(t\right)=\epsilon_{s}\left(t\right)e^{\omega_{s}\left(t\right)},$$

where $\epsilon_{s}\left(t\right)$ and $\omega_{s}\left(t\right)$ are normal processes with zero mean and standard deviations equal to σ(s) and λ(s), respectively.

Based on Equation (1), we can write a probability density function for $\delta_{s}x\left(t\right)$ as:

(2) $$P_{s}\left(\delta_{s}x\right)=\int_{0}^{\infty }G_{s}\left(\ln\sigma \left(s\right)\right)\frac{1}{\sigma (s)}F_{s}\left(\frac{\delta_{s}x}{\sigma (s)}\right)d\left(\ln\sigma \left(s\right)\right),$$

where:

(3) $$\begin{array}{ccc} G_{s}\left(\ln\sigma \left(s\right)\right) & = & \frac{1}{\sqrt{2\pi }\lambda \left(s\right)}\exp\left[-\frac{{\left(\ln\sigma \left(s\right)+\lambda^{2}\left(s\right)\right)}^{2}}{2\lambda^{2}\left(s\right)}\right], \end{array}$$

and:

(4) $$\begin{array}{ccc} F_{s}\left(\frac{\delta_{s}x}{\sigma (s)}\right) & = & \frac{1}{\sqrt{2\pi }}\exp\left[-\frac{\delta_{s}x^{2}}{2\sigma^{2}\left(s\right)}\right]. \end{array}$$

Since $\lambda^{2}\left(s\right)$ is the variance of the log-normal part of the process. This parameter is the key representative measure of the non-Gaussianity of the process; if $\lambda^{2}\left(s\right)\to 0$, the PDF of $\delta_{s}x\left(t\right)$ converges to a Gaussian distribution. An estimation of $\lambda^{2}\left(s\right)$ versus a scale *s* is our way of presenting the effect of large fluctuations over different time scales. Furthermore, for showing the effects of the rare events (in the PDF tails), high order moments of fluctuations of order *q*, denoted by $m_{q}$, can be calculated:

(5) $$m_{q}\left(\delta_{s}x\right)=\langle |\left(\delta_{s}x\right){|}^{q}\rangle ={\left[\int |\delta_{s}{x|}^{q}P_{s}\left(\delta_{s}x\right)d\left(\delta_{s}x\right)\right]}^{\frac{1}{q}}.$$

A large value of $m_{q}$ implies a significant role of rare events. If this is found to be independent of *q*, the process is called monofractal; otherwise, it is called a multifractal [56].

### 2.2. Joint Multifractal Approach: The Bi-MRW Method

Muzy et al. [71,72] proposed the Bivariate Multifractal Random Walk (Bi-MRW) method for analyzing two coupled non-Gaussian stochastic processes ($x\left(t\right)=\{x_{1}\left(t\right),x_{2}\left(t\right)\}$) simultaneously, when the increments of each time series are supposed to be generated by the product of normal and log-normal processes:

(6) $$\delta_{s}x\left(t\right)=\left(\delta_{s}x_{1}\left(t\right),\delta_{s}x_{2}\left(t\right)\right)=\left(\epsilon_{1}^{\left(s\right)}\left(t\right)e^{\omega_{1}^{\left(s\right)}\left(t\right)},\epsilon_{2}^{\left(s\right)}\left(t\right)e^{\omega_{2}^{\left(s\right)}\left(t\right)}\right),$$

in which $\left(\epsilon_{1}^{\left(s\right)},\epsilon_{2}^{\left(s\right)}\right)$ and $\left(\omega_{1}^{\left(s\right)},\omega_{2}^{\left(s\right)}\right)$ have a joint normal PDF with zero mean.

Practically, $\left(\epsilon_{1}^{\left(s\right)},\epsilon_{2}^{\left(s\right)}\right)$ have a covariance matrix:

(7) $$\Sigma_{\left(s\right)}\equiv \left(\begin{array}{cc} \sigma_{1}^{2}\left(s\right) & \Sigma (s)\\ \Sigma (s) & \sigma_{2}^{2}\left(s\right)\; \end{array}\right)$$

where $\Sigma \left(s\right)=\rho_{\epsilon }\left(s\right)\sigma_{1}\left(s\right)\sigma_{2}\left(s\right)$ [71].

The covariance matrix of $\left(\omega_{1}^{\left(s\right)},\omega_{2}^{\left(s\right)}\right)$ is denoted by $\Lambda_{\left(s\right)}$ and called a “multifractal matrix” [71]; it is given by:

(8) $$\Lambda_{\left(s\right)}\equiv \left(\begin{array}{cc} \lambda_{1}^{2}\left(s\right) & \Lambda (s)\\ \Lambda (s) & \lambda_{2}^{2}\left(s\right)\; \end{array}\right),$$

where $\Lambda \left(s\right)=\rho_{\omega }\left(s\right)\lambda_{1}\left(s\right)\lambda_{2}\left(s\right)$ and $\rho_{\omega }\left(s\right)$ is the “multifractal correlation coefficient”. The PDFs of $\left(\epsilon_{1}^{\left(s\right)}\;,\epsilon_{2}^{\left(s\right)}\right)$, and $\left(\omega_{1}^{\left(s\right)}\;,\omega_{2}^{\left(s\right)}\right)$ have the following form:

(9) $$\begin{array}{ccc} F_{s}\left(\epsilon_{1}^{\left(s\right)},\epsilon_{2}^{\left(s\right)}\right) & = & \frac{1}{2\pi \sqrt{\mathrm{Det}(\Sigma_{\left(s\right)})}}\;\exp\left(-\frac{\epsilon_{\left(s\right)}^{T}\cdot \Sigma_{\left(s\right)}^{-1}\cdot \epsilon_{\left(s\right)}}{2}\right) \end{array}$$

(10) $$\begin{array}{ccc} G_{s}\left(\omega_{1}^{\left(s\right)},\omega_{2}^{\left(s\right)}\right) & = & \frac{1}{2\pi \sqrt{\mathrm{Det}(\Lambda_{\left(s\right)})}}\;\exp\left(-\frac{\omega_{\left(s\right)}^{T}\cdot \Lambda_{\left(s\right)}^{-1}\cdot {!}_{\left(s\right)}}{2}\right) \end{array}$$

Therefore, the joint PDF of the fluctuations increment vector $\left(\delta_{s}x_{1},\delta_{s}x_{2}\right)$ is given by:

(11) $$P_{s}\left(\delta_{s}x_{1},\delta_{s}x_{2}\right)=\int d\left(\ln\sigma_{1}\left(s\right)\right)\int d\left(\ln\sigma_{2}\left(s\right)\right)G_{s}\left(\ln\sigma_{1}\left(s\right),\ln\sigma_{2}\left(s\right)\right)\frac{1}{\sigma_{1}\left(s\right)}\frac{1}{\sigma_{2}\left(s\right)}F\left(\frac{\delta_{s}x_{1}}{\sigma_{1}\left(s\right)},\frac{\delta_{s}x_{2}}{\sigma_{2}\left(s\right)}\right).$$

It follows from the above definitions of $G_{s}\left(\ln\sigma_{1}\left(s\right),\ln\sigma_{2}\left(s\right)\right)$ and $F_{s}\left(\frac{\delta_{s}x_{1}}{\sigma_{1}\left(s\right)},\frac{\delta_{s}x_{2}}{\sigma_{2}\left(s\right)}\right)$ that:

(12) $$\begin{array}{ccc} & G_{s}\left(\ln\sigma_{1}\left(s\right),\ln\sigma_{2}\left(s\right)\right)=\frac{1}{2\pi \sqrt{\lambda_{1}^{2}\left(s\right)\lambda_{2}^{2}\left(s\right)-\Lambda^{2}\left(s\right)}} & \\ & \exp\left[-\frac{\lambda_{2}^{2}\left(s\right){\left[\ln\sigma_{1}\left(s\right)+\lambda_{1}^{2}\left(s\right)\right]}^{2}+\lambda_{1}^{2}\left(s\right){\left[\ln\sigma_{2}\left(s\right)+\lambda_{2}^{2}\left(s\right)\right]}^{2}-2\Lambda \left(s\right)\left[\ln\sigma_{1}\left(s\right)+\lambda_{1}^{2}\left(s\right)\right]\left[\ln\sigma_{2}\left(s\right)+\lambda_{2}^{2}\left(s\right)\right]}{2\left(\lambda_{1}^{2}\left(s\right)\lambda_{2}^{2}\left(s\right)-\Lambda^{2}\left(s\right)\right)}\right] \end{array}$$

and:

(13) $$\begin{array}{cc} & F_{s}\left(\frac{\delta_{s}x_{1}}{\sigma_{1}\left(s\right)},\frac{\delta_{s}x_{2}}{\sigma_{2}\left(s\right)}\right)=\frac{1}{2\pi \sqrt{\sigma_{1}^{2}\left(s\right)\sigma_{2}^{2}\left(s\right)-\Sigma^{2}\left(s\right)}}\exp\left[-\frac{{\left[\sigma_{2}\left(s\right)\delta_{s}x_{1}\right]}^{2}+{\left[\sigma_{1}\left(s\right)\delta_{s}x_{2}\right]}^{2}-2\Sigma \left(s\right)\delta_{s}x_{1}\delta_{s}x_{2}}{2\left(\sigma_{1}^{2}\left(s\right)\sigma_{2}^{2}\left(s\right)-\Sigma^{2}\left(s\right)\right)}\right]. \end{array}$$

It can be observed that $P_{s}\left(\delta_{s}x_{1},\delta_{s}x_{2}\right)$ becomes equal to $P_{s}\left(\delta_{s}x_{1}\right)P_{s}\left(\delta_{s}x_{2}\right)$ when Λ(s) and Σ(s) tend to zero.

The *q*-th order moment of fluctuation increments for such two processes at scale *s* can be written as:

(14) $$m_{q}^{joint}\left(\delta_{s}x_{1},\delta_{s}x_{2}\right)=\langle |\left(\delta_{s}x_{1}\right){|}^{q}|\left(\delta_{s}x_{2}\right){|}^{q}\rangle ={\left[\int \int |\delta_{s}x_{1}{|}^{q}{\left|\delta_{s}x_{2}\right|}^{q}P_{s}\left(\delta_{s}x_{1},\delta_{s}x_{2}\right)d\left(\delta_{s}x_{1}\right)d\left(\delta_{s}x_{2}\right)\right]}^{\frac{1}{q}}.$$

## 3. Results

The non-Gaussian parameter $\lambda^{2}\left(s\right)$ and the joint multifractal coefficient Λ(s) at scale *s* are obtained from the integral form of cascading rules in Equations (2) and (11). The best values, for $\lambda^{2}\left(s\right)$ and Λ(s) at scale *s*, are found from the global minimum of the chi-squared, $\chi^{2}$ [73,74]:

(15) $$\chi^{2}\left(\Lambda \left(s\right);\Sigma \left(s\right)\right)=\sum_{\delta_{s}x}\frac{{\left[P_{\mathrm{data}}\left(\delta_{s}x\right)-P_{\mathrm{theory}}\left(\delta_{s}x;\Lambda \left(s\right),\Sigma \left(s\right)\right)\right]}^{2}}{\sigma_{\mathrm{data}}^{2}\left(\delta_{s}x\right)+\sigma_{\mathrm{theory}}^{2}\left(\delta_{s}x;\Lambda \left(s\right),\Sigma \left(s\right)\right)},$$

where $P_{\mathrm{data}}\left(\delta_{s}x\right)$ is the joint PDF computed from data, while $P_{\mathrm{theory}}\left(\delta_{s}x;\Lambda \left(s\right),\Sigma \left(s\right)\right)$ is the theoretical joint PDF proposed in Equation (11). By definition, $\sigma_{\mathrm{data}}^{2}\left(\delta_{s}x\right)$ and $\sigma_{\mathrm{theory}}^{2}\left(\delta_{s}x;\Lambda \left(s\right),\Sigma \left(s\right)\right)$ are the mean standard deviation of $P_{\mathrm{data}}\left(\delta_{s}x\right)$ and of $P_{\mathrm{theory}}\left(\delta_{s}x;\Lambda \left(s\right),\Sigma \left(s\right)\right)$, respectively.

The best value of Λ(s) for the theoretical joint PDF is obtained from the fit of the joint PDF to the data:

(16) $$\begin{array}{ccc} \chi^{2}\left(\Lambda \left(s\right)\right) & = & \sum_{\delta_{s}x}\int d\Sigma \left(s\right)\left(\frac{{\left[P_{\mathrm{data}}\left(\delta_{s}x,s\right)-P_{\mathrm{theory}}\left(\delta_{s}x;\Lambda \left(s\right),\Sigma \left(s\right)\right)\right]}^{2}}{\sigma_{\mathrm{data}}^{2}\left(\delta_{s}x,s\right)+\sigma_{\mathrm{theory}}^{2}\left(\delta_{s}x;\Lambda \left(s\right),\Sigma \left(s\right)\right)}\right), \end{array}$$

**Figure 2:**
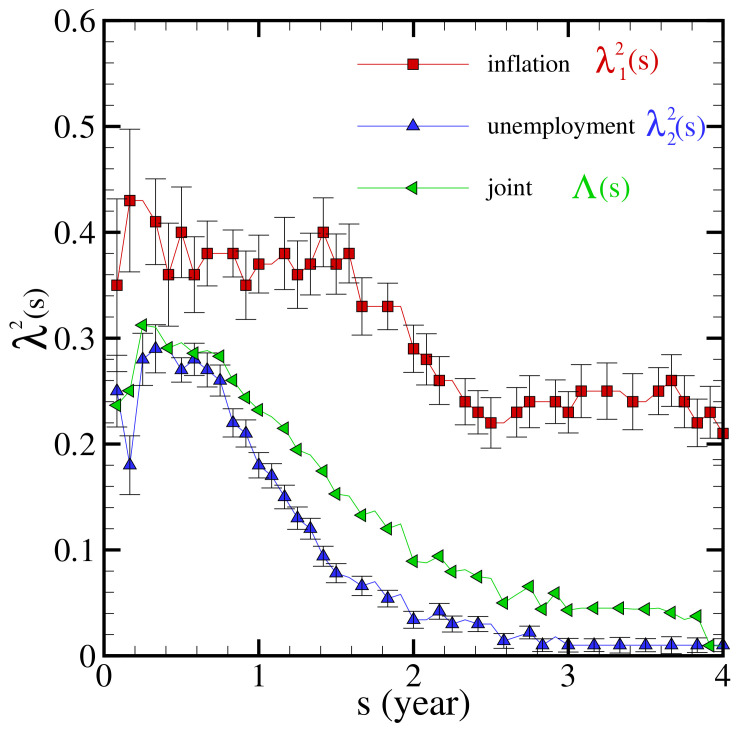
$\lambda^{2}\left(s\right)$ as a measure of the non-Gaussianity for unemployment and inflation index fluctuation distribution and the coefficient Λ(s) for the joint distribution, for different time scales “s”.

The parameter $\lambda^{2}\left(s\right)$ is depicted for inflation, $\lambda_{1}^{2}\left(s\right)$, and unemployment, $\lambda_{2}^{2}\left(s\right)$, in Figure 2. It is seen that $\lambda_{1}^{2}\left(s\right)$ is large at all scales, whereas $\lambda_{2}^{2}\left(s\right)$ is large at scales smaller than one year. Large values of $\lambda_{1}^{2}\left(s\right)$ imply that rare events occurring in the inflation rate make its PDF non-Gaussian. For unemployment, $\lambda_{2}^{2}\left(s\right)$ tends to zero at scales larger than one year. This scaling dependency of $\lambda_{2}^{2}\left(s\right)$ implies that the occurrence of rare events in unemployment provides a non-Gaussian behavior at short time scales, but, after one year, it tends to a Gaussian state.

Concerning the joint multifractal coefficient Λ(s) (see Figure 2), we observe that it has its highest values for scales lower than one year. Thereafter, Λ(s) decreases relatively fast and becomes rather small over the scales over two years. This is compatible with some beliefs about the effect of inflation on the joint relation in the short run and its ineffectiveness in the long run [75].

Next, to improve our understanding of the behavior of the rare events, we turn to higher moments of the variables’ increments. We calculated the moments for various *q* orders and various time scales *s*. Recall that high order moments are much more influenced by the rare events in the tails of the PDF than by small fluctuations.

**Figure 3:**
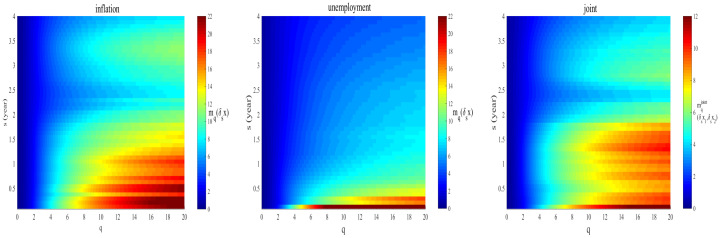
The color map of the inflation or unemployment moment $m_{q}\left(\delta_{s}x\right)$ (from Equation (5)) and joint moment $m_{q}^{joint}\left(\delta_{s}x_{1},\delta_{s}x_{2}\right)$ (from Equation (14)), for different values of *q* and different time scales *s*.

In Figure 3, color intensity plots of high *q* value moments are depicted for different time scales *s*, for inflation or unemployment cases, i.e., $m_{q}\left(\delta_{s}x\right)$, from Equation (5), as well as for the joint distribution. It can be seen that the high moments of the unemployment rates find their largest values for scales below six months. Beyond six months, the moments drop rapidly. In contrast to the unemployment case, the value variation of high moments for the inflation rates is relatively noticeable for all scales.

The right panel in Figure 3 illustrates that the behavior of the joint moment is more similar to the inflation case: a noticeable reduction can be observed for scales above two years. This means that large fluctuations in inflation and unemployment are more strongly coupled above this time interval.

Inflation and unemployment are non-Gaussian series. We know that large events have much more effects on higher moments. The magnitude and intensity of large events in different moments have different effects. Furthermore, since the occurrence of these large events has no specific pattern, we can see a rise and fall in different moments. Therefore, the volatility in the moments is the outcome of these large and unexpected (or rare) events. These rare events could be the effect of political and economic policies.

In the next step, the behavior of the high moments of inflation and unemployment are investigated as time evolved between January 1948 and October 2018. A sliding window of five years was chosen; the moments $m_{q}\left(\delta_{s}x\right)$ were calculated for the one year scale (s=1). The five year window size was chosen since this window size is considered large enough for calculating a meaningful average in such a problem, as in Equations (5) and (14).

Selecting a larger window would flatten the fluctuations, and as a result, the evolution would not be well studied. Moreover, in [22], it was shown that these three variables have different scaling behaviors below and above the five year scale.

**Figure 4:**
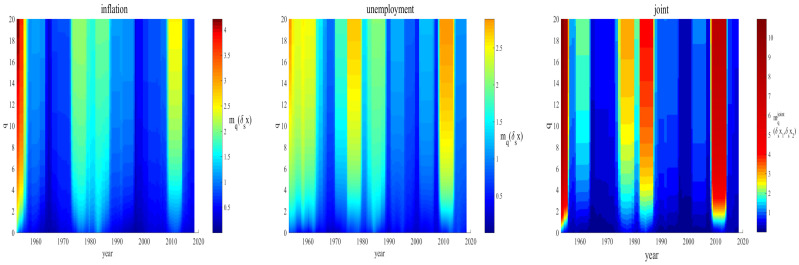
The moments $m_{q}\left(\delta_{s}x\right)$ and $m_{q}^{joint}\left(\delta_{s}x_{1},\delta_{s}x_{2}\right)$ of order *q* are estimated at Scale 1 year from 1948 to 2018 for inflation (**left**), unemployment (**middle**), and the pair of variables (**right**).

In Figure 4, we depict the result for this sliding window. It can be seen, from the red bands, that the joint relation sharply grew at critical periods in the modern history of the U.S. economy, i.e., one can pin the volatile postwar period, the stagflation of the 1970s, the period of the Volcker deflationary program, in the 1980s, and the (recent) Great Recession of 2008–2009.

Moreover, the colors indicate that the inflation rate likely dominates the coupled relation at the post-WWII time; in contrast, the unemployment rate seems to dominate the joint relation in the other historical cases.

## 4. Conclusions

Inflation and unemployment are dependent variables with non-Gaussian PDFs. The scale and intensity of this dependency and their coupling effects have been much debated. The controversy finds its importance when the government aims to impose an expansionary monetary policy over the economic crises. Many researchers have discussed the relation between inflation and unemployment; recall Phillips [1], Friedman [4,5], and many others [6,8,9,10,11,12].

In our work, we focus on the non-Gaussianity of the PDF for the fluctuations of unemployment, inflation, and their coupling. Under the central limit theorem, one should expect that a small fluctuation has a normal distribution. It is however expected that large and rare changes present non-Gaussianity features. Such changes can occur either through unusual market phenomena, such as bubbles and bursts, or through exogenous shocks imposed by a government.

The Multifractal Random Walk (MRW) is known to be a suitable approach to detect the non-Gaussianity of PDFs, through the variance of the log-normal process, i.e., the parameter $\lambda^{2}\left(s\right)$, in Equations (3) and (4). Moreover, the Bivariate Multifractal Random Walk (Bi-MRW) method is useful for analyzing two joint non-Gaussian stochastic processes through the corresponding covariance of Λ(s), Equation (8).

By analyzing 70 years of U.S. data via these techniques, the non-Gaussianity of the PDFs of unemployment and inflation and also that of their joint relation were detected. The non-Gaussianity parameter $\lambda^{2}\left(s\right)$ of the unemployment rate is smaller than that for the inflation and that for the coupled relation Λ(s). It is observed that for scales larger than one year, the behavior of this $\lambda^{2}\left(s\right)$ parameter for the unemployment tends toward a “normal state”; on the contrary, for the inflation data, the non-Gaussianity parameter persists for all studied scales. This means that unexpected fluctuations are observed in a wide range of scales in the inflation phenomenon.

According to Mortensen and Pissarides [76], fluctuations in inflation being larger than the fluctuations in unemployment (we quote) can be attributed to a large extent to differences in policy towards employment protection legislation (which increases the duration of unemployment and reduces the flow into unemployment) and the generosity of the welfare state (which reduces job creation).

Classical economics is interested in long run equilibrium and long run prices. In this school, economists rule out the important role of money value in economic conditions. New Keynesian economics however emphasizes the role of sticky prices. They accept that in the long run, prices are adopted based on the forces in the market. They however claim that if a sudden growth of money value occurs, it takes time for prices to be adjusted to the new values. Therefore, they expect the short-run effects to help policymakers to impose effective stimulation either through a shock in money value or a shock in fiscal stimulation. Interestingly, for policymakers, the non-Gaussianity of the joint relation tends to zero for periods larger than one and up to two years. This means that large and unexpected fluctuations in inflation, either endogenously caused in the market or exogenously imposed by policymakers, have no footprint on the coupling for scales larger than two years. A concrete statement needs further analysis. However, the time window of one to two years might be the time window that divides the short-run and the long-run effects in the market. In other words, for shocks in money volume through the effects of inflation, we observe that the coupling does not inherit such fluctuations in scales above the one to two year time window.

We pointed out that this behavior is further observable in the high-order moments of these (three) variables. Moving the observation window over time, it is discovered that the non-Gaussianities of the parameters $\lambda^{2}\left(s\right)$ and of their coupling Λ(s) substantially grow over the critical (crises) periods of the economy.

In so doing, it seems that we convincingly show that the controversial issue in economics [8,9,10,11,12] about the true relation between unemployment and inflation depends greatly on the considered time scales. This means that policymakers should be flexible and sharply minded, about (their) political horizons, in imposing expansionary monetary policies given boosting production, whence employment, through consumption during and after recessions. Notice that if one still accepts Mortensen and Pissarides’s claims [76], one should wonder whether similar policies should be implemented in Europe and the USA, as well as in other countries. Indeed, if large fluctuations in inflation with respect to unemployment may be ascribed to different degrees of employment protection and the generosity of the welfare state, then this argument may fit quite well some European stories, as compared to the U.S. economy, but one could still argue that this is less convincing in interpreting the joint dynamics of the two variables in the Unites States.

## Abbreviations

The following abbreviations are used in this manuscript:

MRW	Multifractal Random Walk
PDF	Probability Density Function
Bi-MRW	Bivariate Multifractal Random Walk
